# Breastfeeding Continuation at 6 Months among Mother-Infant Dyads Participating in the Postpartum Care in the NICU (PeliCaN) Pilot Trial

**DOI:** 10.1038/s41372-026-02616-x

**Published:** 2026-03-06

**Authors:** Lorelei J. Baumann, Niesha Darden, Rachel Ledyard, Margaret G. Parker, Maggie E. Power, Laura Walker, Celeste P. Durnwald, Heather H. Burris

**Affiliations:** 1 Division of Neonatology, Children’s Hospital of Philadelphia, Philadelphia, PA USA; 2 Department of Pediatrics, UMass Chan Medical School, Worcester, MA USA; 3 Department of Obstetrics and Gynecology, University of Pennsylvania Perelman School of Medicine, Philadelphia, PA USA; 4 Midwifery Program, University of Pennsylvania School of Nursing, Philadelphia, PA USA; 5 Department of Pediatrics, University of Pennsylvania Perelman School of Medicine, Philadelphia, PA USA; 6 Clinical Futures, Children’s Hospital of Philadelphia Research Institute, Philadelphia, PA USA; 7 Leonard Davis Institute of Health Economics, University of Pennsylvania, Philadelphia, PA USA

**Keywords:** Epidemiology, Health policy, Health services

## Introduction

Mother’s milk is the optimal nutrition for preterm infants hospitalized in neonatal intensive care units (NICUs) [1]. Provision of mother’s milk is associated with infant health benefits, including reduced risk of sudden unexpected infant death syndrome and improving neurodevelopmental outcomes [2, 3]. Lactation also has maternal health benefits, particularly for cardiometabolic health and reducing breast cancer risk [3]. Public policy efforts have increased awareness of the importance of mother’s milk, and improved lactation services and insurance coverage for breast pumps. Quality improvement projects have led to increases in mother’s milk provision among very low birth weight (VLBW; ≤1500 g) infants at NICU discharge from 44% in 2008 to 52% in 2017 [1]. However, most infants do not meet the public health goal of exclusive breastfeeding for 6 months, and stark racial disparities in long-term lactation persist. Nationwide data from the Vermont Oxford Network showed that 34.4% of Black infants continued to receive mother’s milk at discharge compared to 56.1% of White infants [1]. Improving breastfeeding through 6 months could mitigate gaps in health outcomes.

Parents prioritize spending time in the NICU and often do not receive comprehensive postpartum care [4]. Our randomized controlled trial (RCT), Postpartum Care in the NICU (PeliCaN), found that embedding doulas and a certified nurse midwife (CNM) in the NICU to provide postpartum support and clinical care, reduced the time to receipt of postpartum care by 20 days and increased rates of depression screening, blood pressure measurement, and contraception counseling [4]. In addition to encouraging maternal healthcare receipt, the PeliCaN doulas and CNM assisted with lactation efforts in the NICU. Therefore, we hypothesized that the PeliCaN intervention would improve breastfeeding rates at 6 months postpartum among participants who initiated lactation, used as a proxy for intent to breastfeed.

## Methods

We conducted a secondary analysis of the PeliCaN RCT [4]. From November 29, 2022, to November 7, 2023, 37 postpartum parents of infants born <34 weeks of gestation at a single tertiary perinatal center in Philadelphia, PA, were eligible to enroll if their infants were <2 weeks of age and anticipated to stay in the NICU for a week minimum. Participants were randomized at a mean (SD) of 5 (2) days postpartum to receive doula support and CNM-delivered postpartum care in the NICU (*n* = 20). Doulas supported participants through emotional bedside presence, breastfeeding education, and feeding equipment logistics. The CNM provided clinical care including latching guidance, milk expression counseling, and coordination with the infant’s medical team regarding safety of medications during lactation. Control participants received usual care, including NICU-provided lactation support (*n* = 17). Any breastfeeding at 6 months postpartum, defined as being fed breastmilk, was a pre-specified secondary outcome assessed with the National Health and Nutrition Examination Breastfeeding Survey, administered remotely via REDCap [5]. The analytic sample was restricted to participants who had initiated lactation prior to enrollment, a marker of intent to breastfeed. Fisher’s exact tests compared proportions of participants who continued to directly breastfeed or express milk at 6 months postpartum. Log-binomial regression models calculated prevalence ratios (PR) of breastfeeding at 6 months, adjusted for parity and infant chronological age at time of survey completion.

## Results

Among the 37 PeliCaN trial participants, 34 completed 6-month follow up surveys (92%, *n* = 17 intervention and *n* = 17 control) (Table 1). Among respondents, 14 (70%) intervention and 15 (88%) control participants reported ever expressing milk prior to trial enrollment. The analytic sample was majority publicly insured (89.7%), identified as Black (75.9%), and worked for pay (65.5%) at 6 months postpartum. Of those who ever expressed milk, 7/14 (50%) intervention participants were still breastfeeding or pumping compared to just 2/15 (13%) of control participants (*p* = 0.05). The association persisted with multivariable adjustment (PR: 4.89, 95% CI: 1.24, 19.3).

**Table 1:** Characteristics of intervention and control participants of the Postpartum Care in the NICU (*PeliCaN*) randomized controlled trial (*n* = 37) among those who both completed a breastfeeding survey at 6-month follow-up (*n* = 34) and initiated lactation prior to enrollment (*n* = 29).

Characteristics	Total (*n* = 29)	Intervention (*n* = 14)	Control (*n* = 15)	*p*-value^a^
**Age (years, mean (SD))**	30.1 (5.8)	29.6 (6.0)	30.6 (5.8)	0.64
**Gestational age at birth**^**b**^				1
<29 weeks	10 (34.5%)	5 (35.7%)	5 (33.3%)	
≥29 weeks	19 (65.5%)	9 (64.3%)	10 (66.7%)	
**Insurance**^**b**^				0.60
Public	26 (89.7%)	12 (85.7%)	14 (93.3%)	
Private	3 (10.3%)	2 (14.3%)	1 (6.7%)	
**Primiparous**	6 (20.7%)	5 (35.7%)	1 (6.7%)	0.08
**Race and Ethnicity**				0.84
Black, non-Hispanic	22 (75.9%)	12 (85.7%)	10 (66.7%)	
Hispanic	4 (13.8%)	2 (14.3%)	2 (13.3%)	
Multiple, non-Hispanic	1 (3.5%)	0 (0.0%)	1 (6.7%)	
Prefer not to answer	1 (3.5%)	0 (0.0%)	1 (6.7%)	
White, non-Hispanic	1 (3.5%)	0 (0.0%)	1 (6.7%)	
**Married or partnered**	20 (69.0%)	8 (57.1%)	12 (80.0%)	0.25
**Cesarean birth**	17 (58.6%)	8 (57.1%)	9 (60.0%)	1
**Neonatal intensive care unit length of stay (months, mean (SD))**	2.7 (2.5)	2.8 (3.2)	2.5 (1.8)	0.72
**Infant age at follow-up (months, mean (SD))**	6.7 (0.6)	6.6 (0.5)	6.9 (0.6)	0.25
**Infant still hospitalized at follow-up**	2 (6.9%)	1 (7.1%)	1 (6.7%)	1
**Working for pay at follow-up**	19 (65.5%)	9 (64.3%)	10 (66.7%)	1
**Breastfeeding**^**c**^ **at follow-up**				0.05
Yes	9 (31.0%)	7 (50.0%)	2 (13.3%)	
No	20 (69.0%)	7 (50.0%)	13 (86.7%)	
Age at discontinuation (months, mean (SD))^d^	3.5 (1.7)	3.0 (1.8)	3.9 (1.7)	0.34

^a^Fisher’s exact, T-test as appropriate.

^b^Stratified randomization factor.

^c^Breastfeeding indicated by ongoing infant feeding of breastmilk.

^d^One intervention and three control participants did not report age at breastfeeding discontinuation.

## Discussion

The PeliCaN model of doula support and CNM-delivered postpartum care in the NICU, after lactation initiation, increased breastfeeding prevalence at 6 months, well after most preterm infants were discharged and intervention concluded. Despite a small sample size overall, it is notable that the majority of participants identified as Black. Given the disproportionate representation of Black infants in NICUs from high preterm birth rates, PeliCaN may serve as a strategy to reduce racial disparities in lactation. We suspect PeliCaN improved breastfeeding through logistical and emotional support. Doulas and midwives focus on supporting wellbeing, infant care confidence, and a sense of connection to feeding goals to help parents feel capable of lactation continuation. Qualitative work and multicentered studies are necessary to inform effectiveness and scalability of PeliCaN to improve breastfeeding for NICU dyads.

## Supplementary information

Consort-2010-Checklist

## Data Availability

The dataset generated for the study is available from the corresponding author on reasonable request and with institutional data use agreements in place.
